# Insights on Personality, Social Support, and Caregiver Burden in Dementia

**DOI:** 10.1002/alz70858_105563

**Published:** 2025-12-26

**Authors:** Christina S Zonker, Aimee R Mooney, Heather Franklin, Allison Lindauer

**Affiliations:** ^1^ Oregon Health & Science University, Portland, OR, USA; ^2^ OHSU School of Nursing, Portland, OR, USA

## Abstract

**Background:**

Previous studies have shown that certain personality traits in family caregivers of persons with dementia are at higher risk for stress, burden and reduced social support. However, the relationship between personality traits, social support, and caregiver burden is not fully understood. Using data from the Tele‐STELLA intervention (R01AG067546), we analyzed whether personality traits influenced caregivers' willingness to contact each other, and if there was an association between contact and burden.

**Method:**

Family caregivers (*N* = 132) participated virtually in an 8‐week psychoeducational intervention to facilitate effective management of behavioral symptoms of dementia. They shared their experiences with other caregivers and were encouraged to contact each other for social support. Personality characteristics were assessed with the Ten‐Item Personality Inventory (TIPI) while stress and burden were measured pre‐ and post‐intervention with the Revised Memory and Behavior Problems Checklist (RMBPC). Post‐intervention, caregivers were queried about contacting another caregiver. Linear and logistic regression models were used for analysis.

**Result:**

Caregivers with TIPI scores higher in agreeableness or lower in openness reported lower burden on the RMBPC (β=‐3.5, *p* = 0.03; β=‐2.2, *p* = 0.04). Lower scores on emotional stability strongly predicted lower burden (β=‐3.7, *p* < 0.001), while higher scores in extraversion were positively associated with higher burden (β=1.7, *p* = 0.05). Higher emotional stability emerged as a significant positive predictor for contacting other caregivers (β=0.51, *p* = 0.02), while higher scores in conscientiousness had a significant negative association on contacting others (β=‐0.7, *p* = 0.02). Whether caregivers contacted each other was not significantly associated with post‐intervention burden score changes.

**Conclusion:**

Results suggest higher scores in agreeableness and lower scores in openness and emotional stability are associated with reduced burden levels. Personality traits such as emotional stability and conscientiousness predicted the likelihood that caregivers would contact other caregivers. Although personality traits were associated with social contact and burden score changes, there was no relationship between seeking support and burden. This suggests that introverted caregivers and those with high conscientiousness may benefit from assistance in identifying peer support, while extroverted caregivers may need additional support in addressing their burden. The findings from this large caregiver sample highlight the need for behavioral studies to consider tailoring interventions to personality typologies.

